# Unilateral and bilateral training competitive archers differ in some potentially unhealthy neck-shoulder region movement behaviour characteristics

**DOI:** 10.1186/s13102-021-00272-6

**Published:** 2021-04-26

**Authors:** Mareike Schmitt, Lutz Vogt, Jan Wilke, Daniel Niederer

**Affiliations:** Department of Sports Medicine and Exercise Physiology, Goethe University Frankfurt am Main, Ginnheimer Landstraße 39, D-60487 Frankfurt am Main, Germany

**Keywords:** Bow, Arrow, Archery, kinematics, Movement behaviour

## Abstract

**Background:**

Excessive unilateral joint loads may lead to overuse disorders. Bilateral training in archery is only performed as a supportive coordination training and as a variation of typical exercise. However, a series of studies demonstrated a crossover transfer of training-induced motor skills to the contralateral side, especially in case of mainly unilateral skills. We compared the cervical spine and shoulder kinematics of unilateral and bilateral training archers.

**Methods:**

In this cross-sectional study, 25 (5 females, 48 ± 14 years) bilaterally training and 50 age-, sex- and level-matched (1:2; 47.3 ± 13.9 years) unilaterally training competitive archers were included. Cervical range of motion (RoM, all planes) and glenohumeral rotation were assessed with an ultrasound-based 3D motion analysis system. Upward rotation of the scapula during abduction and elevation of the arm were measured by means of a digital inclinometer and active shoulder mobility by means of an electronic caliper. All outcomes were compared between groups (unilaterally vs. bilaterally) and sides (pull-hand- vs. bow-hand-side).

**Results:**

Unilateral and bilateral archers showed no between group and no side-to-side-differences in either of the movement direction of the cervical spine. The unilateral archers had higher pull-arm-side total glenohumeral rotation than the bilateral archers (mean, 95% CI), (148°, 144–152° vs. 140°, 135°-145°). In particular, internal rotation (61°, 58–65° vs. 56°, 51–61°) and more upward rotation of the scapula at 45 degrees (12°, 11–14° vs. 8°, 6–10°), 90 degrees (34°, 31–36° vs. 28°, 24–32°), 135 degrees (56°, 53–59° vs. 49°, 46–53°), and maximal (68°, 65–70° vs. 62°, 59–65°) arm abduction differed. The bow- and pull-arm of the unilateral, but not of the bilateral archers, differed in the active mobility of the shoulder (22 cm, 20–24 cm vs. 18 cm, 16–20 cm).

**Conclusions:**

Unilaterally training archers display no unphysiologic movement behaviour of the cervical spine, but show distinct shoulder asymmetris in the bow- and pull-arm-side when compared to bilateral archers in glenohumeral rotation, scapula rotation during arm abduction, and active mobility of the shoulder. These asymmetries in may exceed physiological performance-enhancing degrees. Bilateral training may seems appropriate in archery to prevent asymmetries.

## Introduction

Excessive unilateral joint loads may lead to overuse disorders [1–3]. Archery represents a sport with substantial side dominance. During one single day of competition, approximately 144 arrows are shot [4]. As a result of this, when cumulating the acting loads, an archer pulls over 2.7 tons per day under one-sided conditions [4].

Spinal and pelvic pain in unilaterally training archers are associated with a malalignment of the spine and pelvis, when compared to a healthy non-archery control group [5]. Both malalignements of the spine and pelvis, and asymmetries in the neck-shoulder region movement behavior can be physiological or even performance-enhancing and are not automatically a pathological sign. For example, a glenohumeral internal rotation deficit is only associated with an increased risk for injuries when it exceeds physiological values [6]. In shoulder diseases, the humeroscapular rhythm is often impaired and the rotational movement of the scapula often starts prematurely. Hence, potential malalignments and asymmetries must be rated and cannot be treated as generally harmful.

Hitherto, bilateral training in archery is only performed as supportive coordination training and as a variation to typical shooting practice. However, a series of studies demonstrated a crossover transfer of motor skills from the training to the contralateral side, especially in case of mainly unilateral skills [7, 8]. Bilateral training may consequently represent a performance-enhancing method for the dominant side also in archery. In fact, the assumption of such sport-specific inter-limb transfer is supported by the findings of a parallel group experiment [9]. The study authors reported a bilateral archery training to result in improved dominant-side shooting abilities when compared to conventional unilateral training. Summarizing these two line of thoughts, a bilateral training might be both performance-enhancing and preventive against the development of potentially unhealthy neck-shoulder region musculoskeletal and movement shoulder girdle asymmetries. Although the potential benefits of training bilaterally reach beyond performance-related aspects as it may be associated with reduced asymmetrical joint loading, no study has yet compared the movement behaviour of the cervical spine and the shoulder between unilaterally and bilaterally training athletes. As an experimental proof is hardly realizable due to time and ethical considerations, the present study aimed to address this research deficit adopting a cross-sectional study design. We hypothesize that 1) unilaterally training archers show significant side-to-side asymmetries and 2) that these asymmetries are larger than those in bilaterally training archers.

## Methods

### Study design and ethics

We adopted a matched-cohort cross-sectional design. The study has been reviewed and granted by an independent local ethics committee, all participants subscribed informed consent prior to study inclusion. All research was performed in accordance with relevant guidelines and regulations.

### Participants

Healthy male and female unilateral (no bilateral training experience) and bilateral (bilateral training during at least the previous year) competitive archers aged over 18 years were included. A matched sample with a 2:1 (uni- vs. bilateral) ratio was recruited. Exclusion criteria were: body mass index of > 35 kg/m^2^ [10], pregnancy or nursing period and acute or unhealed injuries of the head, neck, shoulder or upper thorax.

All archers were recruited through word of mouth. The 2:1 matching was done using a standardised matching plan with age, sex, average hours of training per week and the time spent in archery in years as criteria. We screened 29 bilaterally training archery athletes, 25 were included in the study. Subsequently, 760 unilateral archery athletes were screened for matching partner selection, 50 thereof were included and assessed. During screening, potential participants received the same general (unique) information. For the subsequent recruition, a unique standardised consent form was used for all participants.

### Experimental setup and measurements

All outcomes were assessed at one visit. The outcomes consisted of cervical range of motion (RoM), glenohumeral rotational ability, upward rotation of the scapula during abduction and elevation of the arm, and the merging of the fists behind the back. Subsequently, sport-specific data were collected by means of a structured interview and shooting performance was determined. All measurements were performed by one experienced/trained investigator (MS). The standardisation of the participants instructions, the setup and measurement conduction was set and consequently adopted following published recommendations (where applicaple) and, where not available, based on the teams experience. The years of experience in neck and shoulder girdle kinematical assessments with the devices and tests used in the present study of the investigators range between 1 and 23 years.

Cervical RoM was measured using an ultrasound-based 3D motion analysis system, Zebris CMS 70, Zebris Medical GmbH, Isny, Germany. It collects external kinematic data at a sampling rate of 30 Hz. The measurement error of the system is 0.58 ± 1.29 mm, with a relative measurement error of 0.65% [11]. The participants took an upright position on a chair, the hands were positioned on the thighs, and the feet kept on the floor. The mouth remained closed during the measurements. Three markers, mounted on a small, lightweight T-plate, were fixed on the participant’s head with the aid of a helmet-like carrier triplet unit. Another triplet was placed laterally on the lateral aspect of the right upper arm of the participant and served as reference. Head movements were defined as movements of a single, rigid body system with respect to the shoulder embedded coordinate system. Maximal sagittal plane, transverse plane and frontal plane RoMs were assessed. For each direction of movement, three familiarization repeats were followed by five maximum RoM measuring repetitions. All repeats started to the front/right side. A “bull’s-eye” spirit level, positioned on the mask-like carrier unit was used to calibrate the zero position and increase reliability [12].

To estimate glenohumeral rotation capability, the same motion analysis system was used. The t-plate was placed on the medial-distal border of the ulna. Arm rotation movements were defined as movements of a single, rigid body system. Schmidt-Wiethoff et al. [13] found the ultrasound-assisted kinematic analysis of glenohumeral internal and external rotational mobility to be highly reproducible. The standardized sitting position was maintained and the order of measurement of the extremities was determined by prior randomization. The 90° bent arm at the elbow was passively guided at a height of 90° abduction during maximal active internal and external rotation. After a familiarization trial, three measurements were performed. In order to prevent compensatory movements of the shoulder girdle and thoracic spine, the scapula was fixed during the measurement with the aid of the Codmann handle, the humerus was guided passively by the examiner at a height of 90° at the distal end, and motionlessness of the spine was visually controlled.

In order to evaluate the scapulohumeral rhythm, the method of Watson et al. [14], which has been shown to be a clinically reliable approach to measure scapular upward rotation, was used. Exact positioning of the upper arm was ensured by means of the ultrasound-based motion analysis system (marker triplet positioned at the upper arm). A digital inclinometer (Acumar, ACU 360, Lafayette instrument) was attached to the spina scapulae. It served for quantifying upward rotation of the shoulder blade (in degrees). Movement of the scapula in relation to the resting position was recorded at a total shoulder abduction of 45°, 90°, as well as a total shoulder elevation of 135° and the maximum achievable angle. The scapulohumeral rhythm was calculated for each position and then averaged by dividing the shoulder abduction value by the scapular upward rotation value. The intrarater reliability of the measurement method was found to be good [14].

All data assessed with the ultrasound-based motion analysis system were sampled at 100 Hz. All movement behaviour characteristics were calculated from the collected raw 3D ultrasonic movement data. Potential outliers and signal interferences were visually identified and manually filtered.

To measure active shoulder mobility, the participants stood upright, the feet were placed hip-width. They were instructed to move their fists behind their back; the aim was join both fists behind the back. The whole movement had to be performed fluently without interruptions or jerky movements. End-position must be hold for at least 5 s. The movement quality was visually controlled by one of the investigators, only trials fulfilling these two criteria were valid. Each side was tested three times. Outcome was the respective distance between the two fists (cm). The outcome was determined by means of an electronic caliper (ANENG, U.S.A.).

For each of the kinematic outcomes, the best value was selected for further analysis.

The recording of the shooting performance was carried out on a ground-level target (distance of 15 m [9]). This setting was chosen to provide a situation familiar to each archer, as not only target archers (who usually shoot on an 18 m distance and not on ground level) were included, but also 3D-archers (shooting on different distances) and field archers (sometimes shooting downwards). Otherwise, the target archers would have been treated preferentially in view of the shooting performance. The archers used their individual shooting equipment. The target device was a 1 × 1 m disc with a classic five-colour 40 × 40 cm (5 coloured rings but with 10 zones to score) overlay. Before the participants performed 15 shots, they had the opportunity to warm up individually. Six shots were available to get used to the distance and disc position. The remaining nine shots were subsequently scored in three passes of three arrows each. The classification of the individual passes was made according to the rules of the World Archery Federation as a point sum, which resulted from the number of scores shot. All participants performed the shots using their habitual bow and pull sides. In order to make the shooting performance of the individual materials used for shooting comparable, a standardized conversion factor was used [15]. The bows are rated from highest (Compound bow with sight: factor 0.65) to lowest (wooden or “primitive” bow: factor 1.0), the corresponding conversion factor applied to the shooting performance.

### Statistics

Data input, processing, and descriptive analyses were performed using Excel (Microsoft Corporation, 2016, Office 365, Redmond, Washington, USA). The following statistical analyses were performed using SPSS 24 (IBM SPSS, USA).

All values were tested for normal distribution using plots and visual inspection and for variance homogeneity using the Levine’s test. Subsequently, between-group statistics were performed for all participants, archery and training specifics as well as for the self-report outcomes. For that purpose, we performed unpaired t-tests for interval scaled and Chi^2^-tests for ordinal and nominal scaled data. T-Tests were performed in case of variance homogeneity and normal distribution.

For group (unilateral versus bilateral) and side (bow arm/side versus pull arm/side), 2 × 2 repeated measures analyses of variance (rmANOVAS) were performed, main and interaction effects were reported. In case of significant main or interaction effects, post-hoc-comparisons using paired (bow- versus pull-arm) or unpaired (unilateral versus lateral archers) were performed. Group and side-specific data were plotted using arithmetical means and 95-%-confidence intervals (absolute and side-to-side differences).

For all inference statistical tests, a *p*-value of < 5% was considered as significant.

## Results

None of the archers withdrew his/her informed consent, and none had to be excluded. The sociodemographic, physiologic, and archery-specific characteristics of the participants are displayed in Table 1.

**Table 1:** Biometric and archival characteristics of the total and groups comparison, mean ± standard deviation, n = sample size, Match = matching variable; M = Male, F = female; n.s. = not significant

	Total	Unilateral archers	Bilateral archers	Between group difference t-(or Chi) and *p*-value
Number	75	50	25	Match
Sex (m = male, f = female)	M = 60; f = 15	M = 40; f = 10	M = 20; f = 5	Match
Age [years]	47.4 ± 13.9	47.3 ± 13.9	47.6 ± 14.3	Match
Height (m)	1.78 ± 0.1	1.78 ± 0.1	1.78 ± 0.1	n.s.
Body weight [kg)	81.3 ± 12.8	81 ± 12.7	82 ± 13.1	0.1; n.s.
Body mass index (BMI) [kg/m^2^]	25.6 ± 3.4	25.5 ± 3.3	26 ± 3.8	−0.57; n.s.
Archery discipline: Target Archery - Field Archery - 3D Archery [% of participants]	39.2 - 14.5% - .46.3%	38.3 - 17.3% - 44.4%	41.2 - 8.8% - 50%	2.9 n.s.
Archery experience [years]	10.4 ± 7.7	10 ± 7	11 ± 9	−0.53; n.s.
Training frequency [sessions/week]	2.4 ± 1.3	2.4 ± 1.2	2.6 ± 1.6	−0.61; n.s.
Training duration [hours/week]	4.3 ± 2.7	4.4 ± 2.3	4.2 ± 2.9	0.32; n.s.
Secondary upper limb sports [% of particpants]	7	10	5	n.s.
Archery-associated strength / resistance training [% of participants]	22	21	24	n.s.
Eye dominance [% of participants] right – left – unknown	69.3–29.3 – 1.3	74–26 - 0	60–36 - 4	3.0; n.s.
pull-arm-dominance [% of participants] right – left – cross	81.3–18.6 – 21.3	90–10 - 24	64–36 - 16	20.4; < .01
Frequency of participants with a history of cervical spine complaints	15%	14%	16%	0.1; n.s.
Frequency of participants with a history of shoulder complaints	43%	42%	44%	0.1; n.s.
Frequency of participants with a history of trunk complaints	25%	26%	24%	0.1; n.s.
Profession of the archers (mostly sedentary – mostly standing – mostly walking) [% of participants]	68–22 - 10	66–26 - 8	72–14 - 14	5.5; n.s.

### Cervical spine and neck mobility

**Fig. 1:**
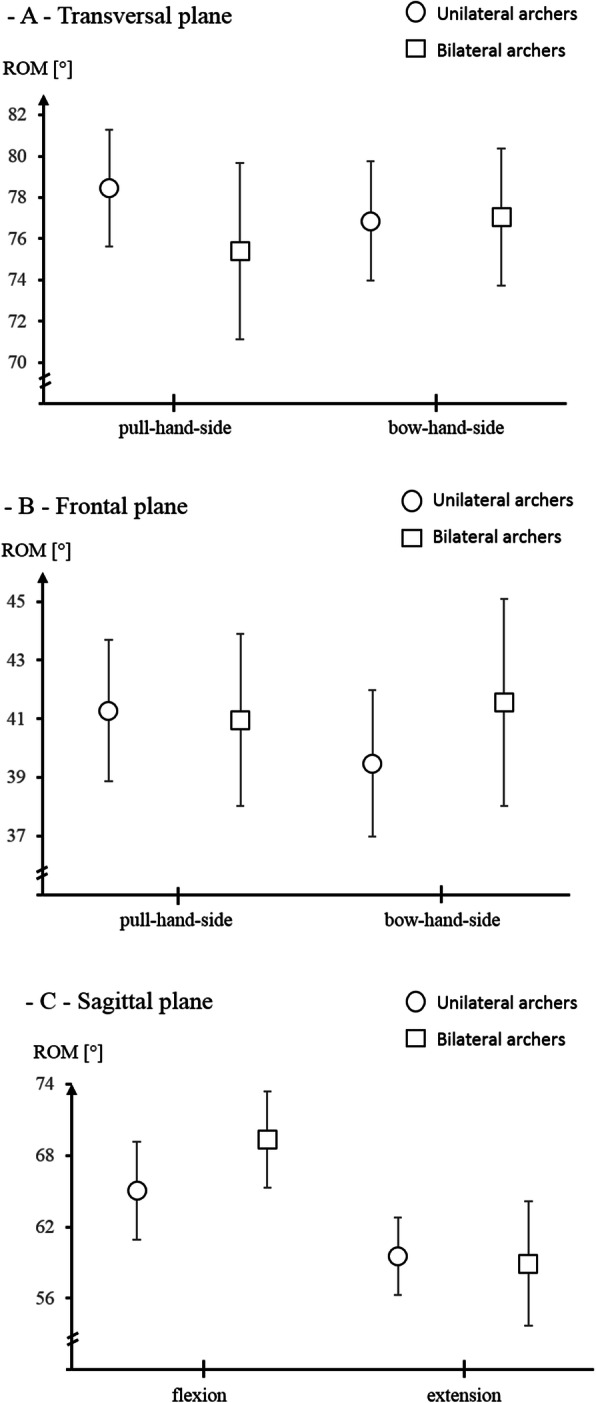
Means and 95% confidence intervals of the cervical range of motion; **a** transversal plane, **b** frontal plane, **c** sagittal plane; ROM = Range of Motion in degree

The cervical RoM showed no significant main between unilateral and bilateral archers effect (transversal plane F = .4, *p* = .5, eta^2^ = .01; frontal plane F = .2, *p* = .7, eta^2^ = .003; sagittal plane F = .7, *p* = .4, eta^2^ = .01), nor a between sides-effect (transversal plane F = .001, *p* = 1, eta^2^ = 0; frontal plane F = .5, p = .4, eta^2^ = .01) or an interaction effect (transversal plane F = 1.9, *p* = .2, eta^2^ = .03; frontal plane F = 2.2, *p* = .1, eta^2^ = .03). The corresponding data are displayed in Fig. 1. Unilateral training archers descriptively displayed a (non-significant) decreased flexion RoM and a decreased transversal plane RoM in the pull hand side when compared to the bilaterally training archers.

### Glenohumeral mobility

For external rotation, the omnibus tests revealed no group (F = 0, *p* = 1, eta^2^ = 0), a significant side (F = 6, *p* = .02, eta^2^ = .08), but no interaction effect (F = 3, *p* = .09, eta^2^ = .04). The internal rotation values showed no significant between-group (F = 2, *p* = .2, eta^2^ = .03), side (F = 3.4, *p* = .07, eta^2^ = .04), or interaction difference (F = 7, *p* = .4, eta^2^ = .01). Total glenohumeral RoM revealed no group (F = 2.7, *p* = .1, eta^2^ = .04), a significant side (F = 6, *p* = .02, eta^2^ = .08), but no interaction effect (F = 3, *p* = .09, eta^2^ = .04).

**Fig. 2:**
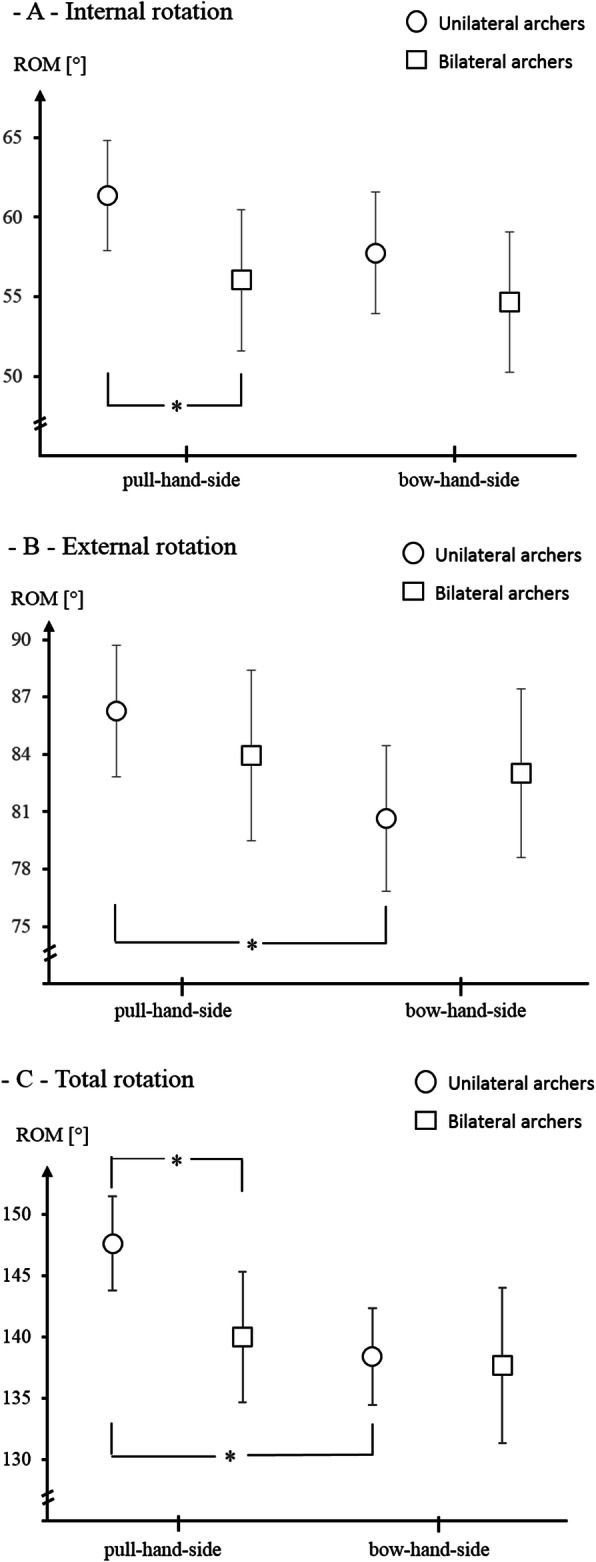
Means and 95% confidence intervals of the glenohumeral rotation mobility, **a** internal rotation, **b** external rotation, **c** total rotation, * = statistically significant differences, RoM = Range of Motion in degree

Post-hoc, the groups differed significantly in internal rotation and total rotation in the pull arm side (unilateral training archers showed larger values, *p* < .05), but not in the bow-hand side (Fig. 2, *p* > .05). The external and total rotation were, in the unilateral training archer only, larger in the pull than in the bow arm side (Fig. 2, both *p* < .05).

### Scapula rotation and scapulo-humeral rhythm

For the upward rotation of the scapula at 45°, the omnibus tests revealed a group (F = 12, *p* = .001, eta^2^ = .2) and side (F = 8, *p* = .006, eta^2^ = .1), but no significant interaction effect (F = 3.5, *p* = .07, eta^2^ = .05). The values for 90° showed a significant between-group (F = 8, p = .006, eta^2^ = .1), no side (F = 3, *p* = .08, eta^2^ = .04), and no interaction difference (F = 2.6, *p* = .1, eta^2^ = .04). At 135°, omnibus test revealed a group (F = 5.3, *p* = .02, eta^2^ = .07), but no side (F = .9, *p* = .4, eta^2^ = .01), or interaction effect (F = .8, *p* = .4, eta^2^ = .01). The values for maximal abduction showed no significant between-group (F = 3.8, *p* = .06, eta^2^ = .05), side (F = 0, *p* = .9, eta^2^ = 0), or interaction difference (F = .9, *p* = .4, eta^2^ = .01).

**Fig. 3:**
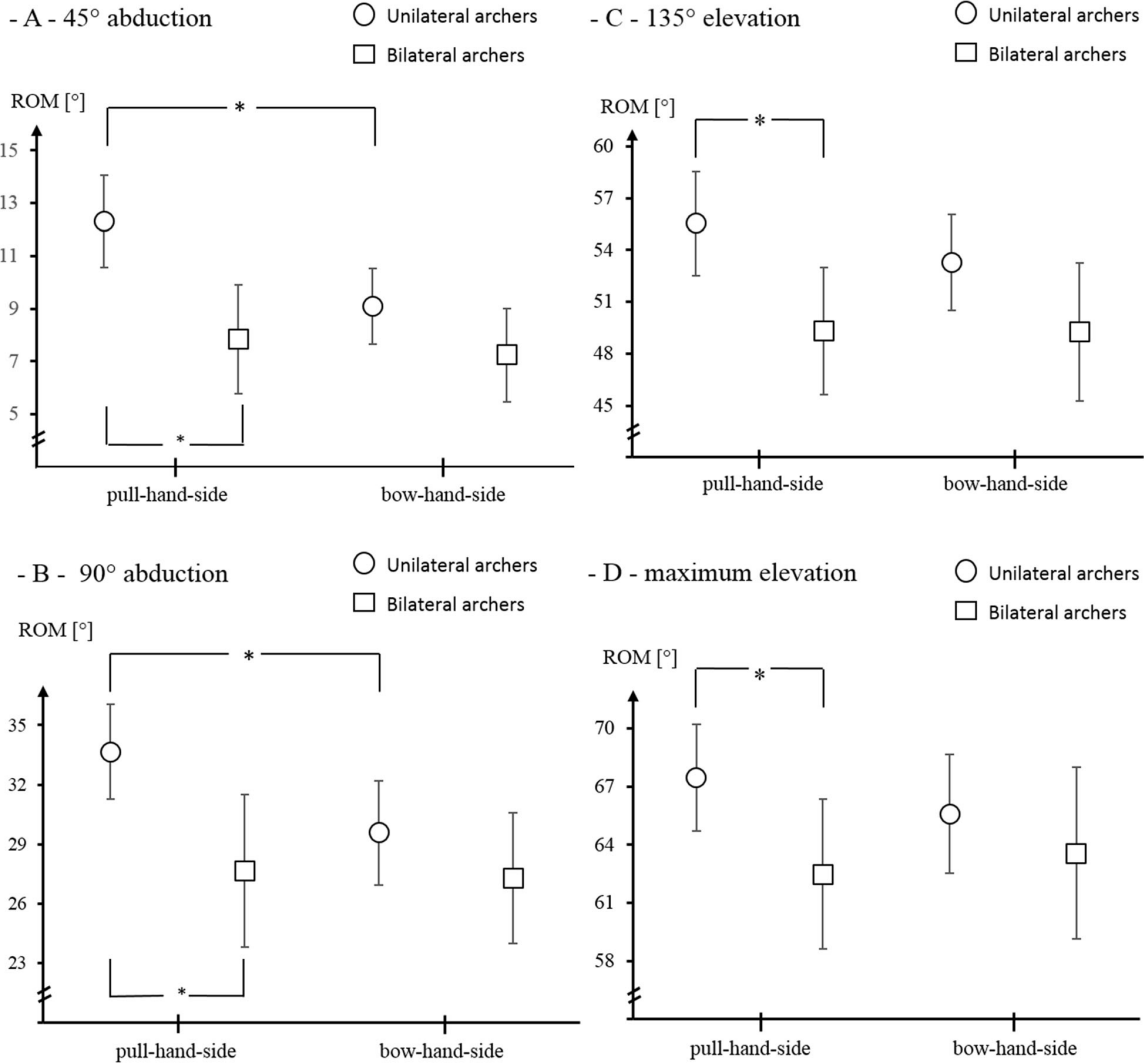
Means and 95% confidence intervals of the upward rotation of the scapula at (**a**) 45°, **b** 90° abduction and (**c**) 135°, **(d)** max. Elevation of the arm, ROM = range of motion in degree, *statistically significant differences

Post-hoc, the groups differed significantly in the upward rotation of the scapula at 45°, 90°, 135° (Fig. 3, *p* < .05). Unilateral training archers showed larger values on the pull hand than in the bow hand side at 45° (Fig. 3, *p* < .05).

The scapulohumeral rhythm differed between the bow-arm- and pull-arm-side of the unilateral archers (ratio of mean 2.9 (± standard deviation 2.1):1 [95% confidence interval 2.3:1–3.6:1] for the pull arm and ratio of 4.2(±2.9):1 [3.40:1–5.1:1] for the bow arm side), but not in the bilateral archers (ratio pull-arm-side 6.1(±5.4):1 [3.9:1–8.3:1] and bow-arm-side 5.3(±3.0):1; [4.1:1–6.6:1]. Between groups, the ratio in the pull-arm-side thus differed significantly (*p* < .05).

### Active shoulder mobility

In the active mobility of the shoulder (distance between the two fists in cm), the omnibus tests revealed no group (F = 0, *p* = .9, eta^2^ = 0), but a side (F = 30, *p* = .001, eta^2^ = .3), and an interaction effect (F = 5.7, *p* = .02, eta^2^ = .07). Post-hoc, the bow- and pull-arm of the unilateral archers differed significantly (bow-arm: mean 22.0 ± standard deviation 7.4 cm [95% Confidence interval: 19.9–24.1 cm]; pull-arm: 18.0 ± 7.7 cm [95% Confidence interval 15.9–20.1 cm]) (each *p* < .05). The shoulder mobility was not different between groups: 1) bow-arm: unilateral archers see above, bilateral archers: 20.9 ± 6.5 cm, [95% Confidence interval 18.4–23.5 cm]; 2) pull-arm: unilateral see above, bilateral archers: 19.3 ± 7.5 cm [95% Confidence interval 16.4–22.3 cm] (each *p* > .05).

### Shooting performance

No differences could be detected in terms of shooting performance (unilateral archers: mean 15.7 ± standard deviation 3.7 scores, [95% confidence interval 14.7–16.7 scores]; bilateral trainings archers: 14.5 ± 4.4 scores, [12.8–16.3], *p* > .05).

## Discussion

When compared to bilaterally training archers, unilaterally training archers exhibit substantial side-to-side shoulder movement asymmetries in glenohumeral rotation, scapula rotation during arm abduction, and active mobility of the shoulder; but not in the movement behaviour of the cervical spine. Our hypotheses 1) and 2) can, thus, only partially be confirmed.

### Shooting performance and pain

In terms of shooting performance, no differences between unilateral and bilateral archers were identified. As a result of matching, both study groups were approximately at a similar level at the time of the measurement when considering average training volume per week and career duration. The archers were not feeling pain at rest or during the tests. That is definitely an important point when functional analyses are performed and rated, as, obviously, pain could have corrupted the movement behaviour during the tests.

### Cervical range of motion

Compared to the general population, both unilateral and bilateral archery athletes had a higher cervical range of motion. The tested athlete’s RoM was about 20° higher than the age-related reference cut-off to distinguish healthy from pathological neck kinematics [16]. The participants may consequently be considered an unimpaired population in view of their neck ranges of motion. The cervical RoM (sagittal plane) was, although not significant, even larger in the bilateral than in the unilateral training archers. As the values exceed published reference values (age-matched) [16], but are still within the confidence range, an unimpaired cervical RoM can be suggested for both groups. In some participants, where the RoM exceeds reference values, the larger RoM could be a sign of higher load and thereby might as well lead to impairments later in life.

### Scapula movement behaviour

Ribeiro and Pascoal [17] noted that asymmetries of a few degrees in the scapula’s movement behavior are not automatically a pathological sign, but rather an adaptation to the sport-specific load and extensive use of the upper limb. On the other hand, the movement behavior of the scapula, especially in athletes from sports that claim the upper limb unilaterally, should not be disregarded. It is essential to clarify the occurrence and potential consequences of sports-specific scapular dyskinesia [17]. According to the current study, unilateral archers show lateral asymmetries in the humeroscapular rhythm of 12 to 27% in 45- and 90-degree abduction position of the upper arm. Bilateral archers differed only by 1 to 8%. Schünke et al. [18] point out that in shoulder diseases the humeroscapular rhythm is impaired and the rotational movement of the scapula often starts prematurely. The causes are manifold and may affect the humeral capsular joint, the subacromial gliding space and the musculature. Due to the contrasting views of an anatomically normal relationship of glenohumeral and scapulothoracic movement in the abduction and elevation of the arm [19, 20], the clinical relevance of the humeroscapular rhythm of our archers can be discussed. As stated above, archery may require a physiologically increased mobility in the shoulder joint of the pull-arm-side due to the posture adopted [21].

### Glenohumeral rotation

So far, there are no comparable studies on the effects of archery on potentially harmful asymmetric movement behaviour. In other one-sided sports, such as baseball, however, sport-specific asymmetries have already been shown [22].

Glenohumeral rotation deficit may be physiological or even performance-enhancing up to a certain point, an association between such deficits and injuries is nevertheless given [6]. It is, overall, unknown if our findings of the side-to-side and unilateral-to-bilateral-archery asymmetries in the shoulder girdle movement behavior are still physiological or already pathological; the latter is commonly defined as a loss of internal rotation greater than 18° and a loss of the total shoulder RoM of greater than 5° [3]. The unilateral group’s in total RoM differs more than 9° between the bow- and pull-arm sides, the bilateral training arches show no difference between sides (in total RoM or internal rotation). Internal rotation (unilateral training) differed only by 4°. However, as the study group of Manske et al. concluded that “A more problematic motion restriction may be that of a loss of (total) ROM in the glenohumeral joint.” and that” Recent evidence supports that a loss of (total) ROM is predictive of future injury to the shoulder in professional athletes.” [3], a pathologically change of the glenohumeral rotation RoM in our unilateral sample is supposable. As we found no difference in shooting performance between unilateral and bilateral training groups and, even more important, a positive interlimb transfer may be given [7, 8] as well in archers [9], it seems tenable that the adaptions of the unilateral archers cannot be rated physiologic (in the sense of being helpful for performance) but rather pathological.

### Physiological mechanism

A possible explanation for the asymmetries between bow- and pull-arm-side in unilateral archers at the upward rotation of the scapula can be found in the activity of the serratus anterior muscle. A dysbalance of the muscle activity of the muscles moving the scapula (reduced serratus anterior and increased upper trapezius activation) may lead to a relative increase in the glenohumeral to scapulothoracal ratio movement during abduction and elevation of the arm [23]. As we have not measured muscle activity, it can only be speculated that this mechanism is existent in our sample. Such a dysbalance would yet be associated with musculoskeletal disorders [23]. The *M. serratus* anterior is responsible for stabilizing the scapula on the bow-arm-side at the beginning of the second movement phase until the shot is released. On the bow-arm-side of the unilateral archers, it is not used in this regard. This fact also seems to explain why bow- and pull-hand-side of bilaterally trained archers, unlike unilateral, do not differ significantly in humero-scapular rhythm. Given the unilateral archery asymmetries in scapulohumeral motor behaviour, bilateral exercise appears to be beneficial from a health perspective. In particular, on the basis of the fact that the *M. serratus* anterior belongs to the local stabilizers of the scapula [24], strengthening on both sides may contribute to shoulder health. Contrary, other mechanisms are supposable: The assymetries could be a direct effect of the joint positioning during shooting; the bilateral shooters spend less time in such extreme positions, the adaptations thus may be less.

### Methodological considerations

Wilke et al. [12] showed that the use of a “bulls-eye” spirit level to calibrate the zero position allows a highly reliable ultrasound-based measurement of cervical movements in the half-cycle. Therefore, the cervical RoM measurement within this study is considered reliable. With the help of the Codmann handle, a co-movement of the scapula was avoided during the measurement of the glenohumeral rotation capability, whereby the measured values are classified as valid [13]. The inclusion criterion for the bilateral participants was a period of bilateral training of at least 1 year. The fewest archers, however, did the bilateral training directly with the entry into the archery. In group matching, only the entirety of the archery years was taken into account. This was an average of 11.2 years for the participants classified as bilateral, whereas on average, however, they had only been practicing archery bilaterally for 4.6 years. Since the number of bilaterally trained archery athletes is small, a different approach was not possible. As our study design was quasi-experimental and not experimental (which is almost impossible to be applied), the association and differences, although supposable effects of the uni- vs. bilateral training, may also be influenced by unknown confounders.

## Conclusion

Unilaterally training archers display no unphysiologic movement behaviour of the cervical spine, but show distinct shoulder asymmetris in the bow- and pull-arm-side when compared to bilateral archers in glenohumeral rotation, scapula rotation during arm abduction, and active mobility of the shoulder. These asymmetries may lead to an increased injury and disorder risk and should be investigated in upcoming research. As a consequence of the potential adverse effects of unilateral training and in view of the performance-enhancing impact of bilateral shooting, archery coaches may be encouraged to consider the integration of shooting with the non-dominant side in training.

## Data Availability

The datasets used and/or analysed during the current study available from the corresponding author on reasonable request.

## Abbreviations

RoM: Range of motion
CI: Confidence interval
